# The Friedmann Anti-Tuberculosis Serum

**Published:** 1913-03

**Authors:** 


### The Friedmann Anti-Tuberculosis Serum

Our readers have been informed as to the pros and cons of this subject through the daily press. We defer any expression of opinion as to its merits for a couple of years at least. With a natural scepticism due to past experience in many failures of analogous cures, certain diagnostic signs, etc., we confess to a general optimism. Some time, some one, somehow, will show us how to eradicate tuberculosis. It may be by the patient application of a number of prophylactic and hygienic measures or by some one brilliant method of therapeutics or specific prophylaxis. Perhaps the tubercular millenium has been reached, perhaps not,

But we want to draw a moral, apropos of the agitation regarding animal experimentation, and of the present tendency to establish institutions of research along all scientific lines. It should be recognized that men of heterodox opinions, apparently lacking in preliminary qualifications, and hopelessly lacking in influence, have in spite of—or perhaps because of—these circumstances, contributed much to science, medical and general. Absolutely nothing in the way of restrictive legislation or of a closed door policy, should be tolerated to the discouragement of the independent worker.

And, at some risk of being misunderstood, to imply more than is intended, we wish to say just a word as to the condemnation of Dr. Friedmann on the ground of commercializing his discovery. There is a very ancient rule of ethics, on good authority, to the effect that the Laborer is worthy of his hire. Always provided that one is sincere and honorable, valuable labor is worth high wages. With these qualifications, we can see no more objection to taking advantage of a notable discovery than of social and professional standing, to charge an unusually high fee. And, while the man who merely increases his schedule of charges for routine work because of social and professional prestige chiefly benefits himself and tends to shorten his period of active usefulness by the ability to retire early, the man who has made a great discovery increases his efficiency to humanity by making himself independent of sordid cares and distractions. These remarks do not, however, apply to the man who simply grasps at the opportunity afforded by a temporary and fictitious notoriety.

Dr. Hesse of Berlin will contribute a report on this subject in our next issue.

